# Acupuncture Is Effective at Reducing the Risk of Stroke in Patients with Migraines: A Real-World, Large-Scale Cohort Study with 19-Years of Follow-Up

**DOI:** 10.3390/ijerph20031690

**Published:** 2023-01-17

**Authors:** Chung-Chih Liao, Chi-Hsien Chien, Ying-Hsiu Shih, Fuu-Jen Tsai, Jung-Miao Li

**Affiliations:** 1 Department of Post-Baccalaureate Veterinary Medicine, Asia University, Taichung 41354, Taiwan; 2 Management Office for Health Data, China Medical University Hospital, Taichung 40201, Taiwan; 3 College of Medicine, China Medical University, Taichung 40402, Taiwan; 4 School of Chinese Medicine, College of Chinese Medicine, China Medical University, Taichung 40402, Taiwan; 5 Department of Medical Research, China Medical University Hospital, China Medical University, Taichung 40402, Taiwan; 6 Division of Medical Genetics, China Medical University Children’s Hospital, Taichung 40447, Taiwan; 7 Department of Biotechnology and Bioinformatics, Asia University, Taichung 41354, Taiwan; 8 Department of Chinese Medicine, China Medical University Hospital, Taichung 40447, Taiwan

**Keywords:** migraine, stroke, acupuncture, National Health Insurance Research Database

## Abstract

Migraines are common headache disorders and risk factors for subsequent strokes. Acupuncture has been widely used in the treatment of migraines; however, few studies have examined whether its use reduces the risk of strokes in migraineurs. This study explored the long-term effects of acupuncture treatment on stroke risk in migraineurs using national real-world data. We collected new migraine patients from the Taiwan National Health Insurance Research Database (NHIRD) from 1 January 2000 to 31 December 2017. Using 1:1 propensity-score matching, we assigned patients to either an acupuncture or non-acupuncture cohort and followed up until the end of 2018. The incidence of stroke in the two cohorts was compared using the Cox proportional hazards regression analysis. Each cohort was composed of 1354 newly diagnosed migraineurs with similar baseline characteristics. Compared with the non-acupuncture cohort, the acupuncture cohort had a significantly reduced risk of stroke (adjusted hazard ratio, 0.4; 95% confidence interval, 0.35–0.46). The Kaplan–Meier model showed a significantly lower cumulative incidence of stroke in migraine patients who received acupuncture during the 19-year follow-up (log-rank test, *p* < 0.001). Acupuncture confers protective benefits on migraineurs by reducing the risk of stroke. Our results provide new insights for clinicians and public health experts.

## 1. Introduction

Migraines are common neurovascular disorders affecting more than 1 billion people worldwide and are the second most disabling disease globally [1,2]. In addition to the unbearable unilateral, throbbing headache, a migraine may also be accompanied by symptoms, such as nausea and/or vomiting or photophobia and/or phonophobia, which often seriously affect a person’s quality of work and life [3]. Currently, migraines are mainly treated with pharmacological therapy. The treatment goal is to relieve the pain symptoms of acute headaches, and reduce the frequency of attacks and rate of recurrence, thus enabling the patient’s return to normal work and life as quickly as possible [4]. However, some patients have poor tolerance to oral medications, and the unpleasant side effects and lower-than-expected efficacy of these medications may lead to low treatment compliance and other complications, causing some patients to choose acupuncture therapy [5].

Strokes, including ischemic and hemorrhagic strokes, are the leading causes of death and disability worldwide. According to one survey, the global lifetime risk of a stroke at age 25 and older is about 25% in both men and women [6]. Even if a patient survives a stroke, he/she is usually left with some degree of neurological deficit, which often imposes a heavy life and financial burden on both patients and their caregivers.

There is growing evidence that migraines increase the risk of strokes. A migraine with aura is considered a risk factor for ischemic stroke, while high frequency and recent onset of migraine are also associated with an increased risk of ischemic stroke [7]. A nationwide population-based study concluded that migraines are associated with an increased risk of ischemic stroke, especially in young (age ≤ 45 years) women with migraines with aura [8]. In another recent study, both a migraine without aura and a migraine with aura were associated with an increased risk of a stroke compared with non-migraineurs (adjusted hazard ratio (aHR) 1.49 and 1.63, respectively) [9]. However, there is currently no direct evidence to support that the preventive medication treatment recommended for use reduces future stroke attacks in migraineurs [7,10].

Acupuncture therapy is one of the main treatment techniques of traditional Chinese medicine. It has a history spanning more than 3000 years and is now widely used in countries all over the world. Acupuncture is considered to be an effective alternative therapy for migraines [11]. An overview of systematic reviews concluded that acupuncture leads to a higher reduction in the number of days with headaches and the use of pain medication, and is more effective at reducing headache frequency and severity, than the same parameters associated with Western medicine and sham acupuncture [12]. A randomized clinical trial concluded that acupuncture treatment was more efficient than sham acupuncture or no acupuncture treatment in reducing long-term recurrent migraine in patients with migraines without aura [13]. Furthermore, in one study, patients in the group receiving real acupuncture were significantly better at controlling pain levels and improving quality of life with chronic migraines than those in the sham acupuncture group [14]. Another study revealed that preventive acupuncture in patients with chronic migraine significantly reduced the average number of moderate/severe headache days per month compared with the change associated with topiramate treatment [15]. In addition, a growing number of clinical trials have shown that acupuncture, when used in stroke therapy, provides excellent complementary and alternative therapy for post-stroke rehabilitation; it also serves as a preventive strategy that induces cerebral ischemic tolerance [16,17,18,19]. Therefore, we investigated whether acupuncture could provide long-term protection against strokes in patients with migraines.

To date, there has been no large-scale real-world evidence to show whether acupuncture is beneficial in reducing the risk of subsequent strokes in migraineurs. Therefore, we conducted a cohort study using a national database to explore the relationship between acupuncture intervention and stroke development in patients with migraines.

## 2. Materials and Methods

### 2.1. Data Source

The study used the Longitudinal Generation Tracking Database 2005 (LGTD 2005) for a nationwide, population-based cohort analysis. The dataset contained all claims data from a random sample of 2,000,000 from the National Health Insurance Research Database (NHIRD) in Taiwan. In LGTD 2005, in order to protect personal privacy, scrambled, anonymous identification numbers were used, and detailed medical information related to traditional Chinese medicine and western medicine was recorded, such as demographic characteristics, medical visits, doctor’s diagnosis, prescription drugs, surgical procedures, and medical expenditure from 2000 to 2018. The International Classification of Diseases, Ninth Revision and Tenth Revision, Clinical Modification (ICD-9-CM and ICD-10-CM) was used for the diagnosis codes. The Institutional Review Board of the China Medical University in central Taiwan (CMUH110-REC1-038(CR-1)) approved this study.

### 2.2. Study Population

**Figure 1:**
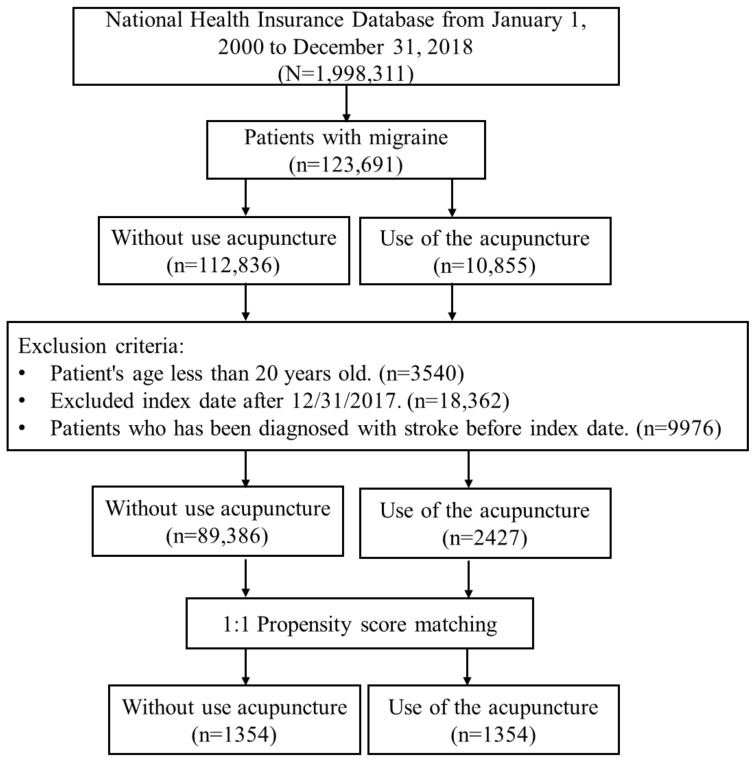
Study flowchart. LGTD 2005: Longitudinal Generation Tracking Database 2005.

We enrolled all participants newly diagnosed with migraines (ICD-9-CM: 346 or ICD-10-CM code: G43.109, G43.501, G43.509, G43.601, G43.609, G43.101, G43.111, G43.119, G43.511, G43.519, G43.611, G43.619, G43.009, G43.011, G43.019, G43.711, G43.719, G43.801, G43.809, G43.A0, G43.B0, G43.C0, G43.D0, G44.001, G44.009, G44.011, G44.019, G44.021, G44.029, G43.811, G43.819, G43.A1, G43.B1, G43.C1, G43.D1, G43.401, G43.409, G43.411, G43.419, G43.701, G43.709, G43.901, G43.909, G44.031, G44.039, G44.041, G44.049, G44.51, G43.911, G43.919), with at least two outpatients or hospitalizations from 1 January 2000, to 31 December 2018, in the LGTD 2005 (n = 123,691). Patients who received acupuncture after the new migraine diagnosis date were defined as acupuncture users, while the remaining patients were defined as acupuncture non-users. In addition, the date of receiving the first acupuncture treatment after the new diagnosis date of the migraine was defined as the index date for the acupuncture cohort. Regarding the index date for the acupuncture non-users, we randomly assigned a month and day to the same index year of the matched acupuncture cases. We excluded subjects aged < 20 years (n = 3540), index date after 31 December 2017 (n = 18,362), and those with a history of stroke (ICD-9-CM 430–438 or ICD-10-CM: I60-I69) before the index date (n = 9976). The 1:1 propensity score method by sex, age, urbanization level, occupation, monthly income, baseline comorbidities, and medication was used to match an equal number of patients in both cohorts. The research process is shown in Figure 1. The primary outcome of the study was the incidence of stroke (ICD-9-CM 430-438 or ICD-10-CM: I60-I69), as diagnosed by a nationally certified neurologist. The follow-up period in both cohorts started from the index date until diagnosis with stroke, death, withdrawal from the National Health Insurance (NHI) program, or the end of 2018. Baseline comorbidities were considered to exist, including diabetes mellitus, hypertension, hyperlipidemia, coronary artery disease, head injury, Parkinson’s disease, chronic kidney disease, and mental disorders. Medication usage was defined as the first prescribed medication after migraine and included triptans, ergots, acetaminophen, NSAIDs (including etoricoxib, celecoxib, and ibuprofen), propranolol, flunarizine, anti-epileptics (including valproic acid and topiramate), and amitriptyline. Patient medication usage was classified (into groups) as those taking 0–1, 2–3, or >3 medications, according to the accumulated number of medications prescribed throughout the follow-up.

### 2.3. Statistical Analysis

Differences in baseline characteristics between both groups were examined using the standardized mean difference (SMD). A standardized mean difference of 0.1 or less indicates a negligible difference. The incidence density rates of strokes were calculated for both acupuncture and non-acupuncture cohorts. Univariable and multivariable Cox proportional hazard regressions were performed to evaluate the hazard ratios (HRs) with 95% confidence intervals (CIs) for strokes. The Kaplan–Meier method was used to estimate the differences in the cumulative incidence of strokes between the two cohorts by the log-rank test. A *p*-value of less than 0.05 indicated statistical significance. All statistical analyses and figures were performed using SAS software, version 9.4 (SAS Institute, Inc., Cary, NC, USA), and survival curves were drawn using R software (R version 4.2.0).

## 3. Results

The study randomly selected 1354 patients in the acupuncture and non-acupuncture cohorts using the 1:1 propensity score-matching method. Table 1 presents the baseline characteristics of both cohorts. In terms of sex, age, urbanization level, occupation, income, baseline comorbidities, and medication use, there were no significant differences between the cohorts. The ratio of females to males was about 7:3. Regarding the age subgroups, the highest proportion of migraine sufferers in both groups was in the 40–59 age group, with a mean age of about 47 years. The most common baseline comorbidities in both groups were mental disorders (44%), followed by hypertension (25%), and hyperlipidemia (14%). Most migraine sufferers (52%) took two to three different medications. The average follow-up time for stroke was 13.77 years in the acupuncture cohort and 9.71 years in the non-acupuncture cohort.

Table 2 shows the crude and adjusted HRs for stroke and covariates in migraineurs. Among patients with migraines, 387 patients (20.76 per 1000 person-years) who received acupuncture and 636 patients (48.38 per 1000 person-years) who did not receive acupuncture had strokes during the follow-up. In a Cox proportional hazards model, after adjusting for sex, age, urbanization level, occupation, income, baseline comorbidities, and medication use, migraineurs receiving acupuncture had a significantly lower risk of strokes than acupuncture non-users (aHR, 0.4; 95% CI, 0.35–0.46). There was no significant difference in the risk of stroke development between males and females with migraines (males vs. females: aHR, 1.02; 95% CI, 0.88–1.17). Among patients with migraines, the risk of a stroke exhibited a dose-dependent increase with advancing age (aHR, 2.55; 95% CI, 2.1–3.09 for patients aged 40–59 years vs. 20–39 years; aHR, 4.93; 95% CI, 4–6.09 for patients aged >59 years vs. 20–39 years). Hypertension, coronary artery disease, head injury, Parkinson’s disease, and mental disorders were baseline comorbidities that increased the risk of a stroke. Patients with migraines who took more medications had a dose-dependent lower risk of a stroke (aHR, 0.18; 95% CI, 0.15–0.21 for 2–3 medications vs. 0–1 medication; aHR, 0.11; 95% CI, 0.09–0.13 for >3 medications vs. 0–1 medication).

Table 3 displays the incidence rates and HRs of strokes in acupuncture users compared to acupuncture non-users stratified by sex, age, urbanization level, occupation, income, baseline comorbidities, and medication use. The beneficial effect of receiving acupuncture treatment on stroke incidence was observed in both female and male migraine patients (aHR, 0.37; 95% CI, 0.32–0.43 for females; aHR, 0.32; 95% CI, 0.25–0.41 for males). Acupuncture reduced the risk of strokes in patients in all age groups and in patients who did or did not have baseline comorbidities. Patients in the acupuncture cohort using any drug doses were less likely to have strokes than those in the non-acupuncture cohort.

We further compared the incidence rate of stroke for ischemic and hemorrhagic strokes. The results in Table 4 show that, compared with non-acupuncture patients, patients who received acupuncture had a lower risk of these types of strokes.

**Figure 2:**
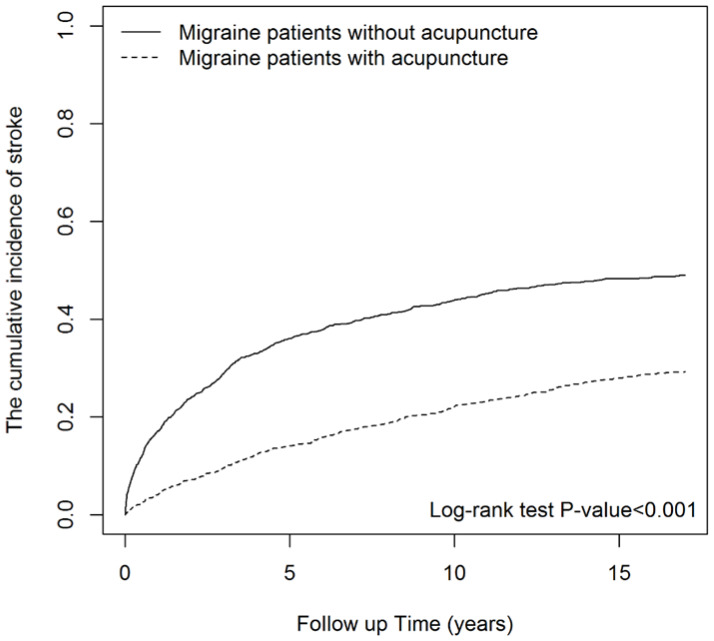
Cumulative incidence of strokes in the acupuncture and non-acupuncture cohorts.

Figure 2 shows that—using the Kaplan–Meier analysis—the cumulative incidence of strokes in patients with migraines was significantly lower in the acupuncture users than in the acupuncture non-users during the 19-year follow-up period (log-rank test, *p* < 0.001).

## 4. Discussion

Our study attempted to use a large real-world dataset with long-term follow-up to determine whether acupuncture treatment could significantly reduce the risk of stroke development in migraineurs. The results of the study found that acupuncture treatment reduces the risk of strokes in patients with migraines by approximately 60%, showing that this treatment method has a good protective effect against strokes.

The Taiwanese government started creating the country’s NHI program in 1995 to cover the entire public insurance system. At present, NHI coverage reaches more than 99% of the 23 million residents of Taiwan. The NHI program includes both Western and traditional Chinese medicine and provides representative and empirical health insurance data for use in research [20,21]. The Central Health Insurance Bureau has been building the NHIRD since 1998 and has provided value-added services for its data to facilitate related medical and public health research since 2000. The NHIRD provides a large population sample: real-world evidence that can eliminate bias from limited sample sizes. In addition, the database presents a long-term follow-up window of more than 10 years, which makes it easier for researchers to study the development of chronic diseases and the risk of related diseases. In Taiwan, traditional Chinese medicine treatment, which includes acupuncture, is only performed by qualified traditional Chinese medicine doctors after complete training and national certification. In clinical practice, this modality is completely complementary to modern medicine and ensures that patients are provided with the most suitable diagnoses and treatments. The relevant treatment records are included in the NHIRD. Therefore, we consider this database to be a trustworthy research tool from which we developed unbiased real-world evidence for our study.

We found no difference in stroke risk between women and men. This appears to contradict many previous studies on migraines and strokes, which reported a predominance of stroke risks in women [10,22]. We speculate that several reasons are behind this. Most studies report that female migraine patients are more prone to ischemic strokes than their male counterparts, but the relationship with hemorrhagic stroke remains controversial [23,24,25,26]. However, our study evaluated all types of strokes, which might have influenced our results. The prevalence of migraines in men is approximately one-third that of women, and the association with strokes is more uncertain in men than in women [27], especially in our limited paired sample. In addition, we did not include migraine-free male and female populations with strokes for comparison, which might also have led to different results. However, acupuncture was associated with reduced stroke risk in both male and female patients with migraines. Patients with migraines in the present study who were aged 40–59 years and >59 years had a higher risk of strokes than younger populations, suggesting that the risk of a stroke increases with increasing age. This finding is similar in general populations without migraines [28,29], indicating that aging may be a pivotal factor that increases the incidence of strokes in the migraine population. We found that acupuncture significantly reduced the risk of strokes in all age groups of patients with migraines and that patients of older ages had a higher risk of strokes. Thus, we infer that acupuncture treatment offers a very good and substantial protective benefit to older patients with migraines.

In our study, among the listed comorbidities that were associated with migraines, hypertension, coronary artery disease, head injury, Parkinson’s disease, and mental disorders were independently found to significantly increase the risk of stroke. Hypertension is the most common risk factor for stroke, high blood pressure can increase the risk of strokes by four times, and routine blood pressure management is very important [30,31]. Previous studies have shown that a substantial proportion of stroke patients have preclinical coronary artery disease and that there is a clear relationship between cerebral and coronary atherosclerosis, both in terms of location and burden [32]. A meta-analysis showed that traumatic brain injury was an independent risk factor for strokes, regardless of the severity or type of trauma [33]. A nationwide cohort study found that the head injury group had a significantly higher stroke risk than the control group (adjusted HR 1.65). Moreover, in the head injury group, the cumulative incidences of ischemic and hemorrhagic strokes were higher than those in the control group (8.9% vs. 5.8% and 2.7% vs. 1.6%, respectively), indicating that a head injury is an independent risk factor for ischemic and hemorrhagic strokes [34]. Similarly, another study calculated an HR of 2.37 for strokes in the Parkinson’s disease group compared with the non-Parkinson’s disease group during 3 years of follow-up, indicating that patients with Parkinson’s disease have a significantly increased risk of ischemic stroke [35]. A meta-analysis of 17 prospective studies involving 206,641 participants showed a significant positive association between depression and the subsequent risk of a stroke after adjusting for potential confounders [36]. Additionally, in a systematic review and meta-analysis of eight studies including 950,759 patients, researchers observed a 24% increased stroke risk in people with anxiety disorders compared to the general population [37]. We found that acupuncture could potentially reduce the incidence of stroke in patients with these underlying comorbidities. Many clinical and experimental studies have been published that show the benefits of acupuncture treatment for treating hypertension [38,39], coronary artery disease [40,41], head injury [42,43], Parkinson’s disease [44,45], and mental disorders [46,47]. Since the above-mentioned potential comorbidities of migraines increase the risk of strokes, and acupuncture shows an independent benefit for patients with these comorbidities, we can consider acupuncture as a treatment that can reduce the risk of strokes in patients with migraines, perhaps by aiding in the control of any accompanying comorbidities. Moreover, while no existing preventive medication for migraines reduces future strokes, our study found that participants who took more medications for migraines had a lower risk of stroke. This result deserves further clarification and may be related to the fact that patients who took more medication, either because they experienced more pain or because they had less control, were likely to return more often for medical advice and to make more aggressive changes to their diets and lifestyles. The habit of taking care of the body in this way may prevent strokes and other cerebrovascular diseases. Nevertheless, we determined that regardless of the quantity of migraine medication taken, it has a positive effect on stroke prevention when combined with acupuncture.

Although the mechanisms by which migraines increase the risk of strokes are not fully understood, several associated pathological mechanisms have been discovered. One study suggested that migraines may directly cause migraine infarction by inducing cerebral microcirculatory vasoconstriction, cerebral large vessel spasms, and vascular endothelial-related hypercoagulability [48]. Another study mentioned that the hypoperfusion of the brain that occurs during a migraine attack can lead to electrical abnormalities that cause a phenomenon called “spreading depression”, which can lead to strokes [49]. Acupuncture treatment has been proven beneficial in the clinical treatment of migraines [50,51], and in effectively reducing stroke risks in patients with depression [18], and fibromyalgia [52]. Since migraines and strokes are heterogeneous cerebrovascular diseases with common mechanisms, it is reasonable to hypothesize that when acupuncture is used to treat patients with migraines, it would be beneficial in reducing the risk of a stroke.

Our present study clearly illustrates that acupuncture complements modern medical treatment to bring about a reduced incidence of stroke development in patients with migraines. The question then arises of how acupuncture is able to achieve such an effect. In the future, more basic experiments should be conducted to clarify the mechanism of action of acupuncture to support our clinical data.

Our study had several limitations. (1) The effect of acupuncture treatment on the risk of strokes in migraine patients of different degrees was not explored. Quantifications of actual severity and frequency of headache attacks in migraineurs were not recorded in the NHIRD medical records. However, the subcategories of migraine ICD codes, such as refractory migraine, non-refractory migraine, episodic migraine, and chronic migraine, could guide further research on this topic. In addition, we did not clarify the association between the number, frequency, timing, and duration of acupuncture treatment in patients with migraines and the risk of a stroke. Future studies could examine the differences in the risk of strokes among patients receiving acupuncture for the first time versus those who have tried other medications, or compare the stroke risk of patients with acupuncture–refractory migraines versus the stroke risk of patients with non-refractory migraines. Furthermore, aura is a clear risk factor for strokes in migraineurs [7]. We did not divide patients with migraines into subgroups with and without aura. Therefore, studies including larger datasets are necessary to test whether acupuncture leads to an overestimation of stroke risk reduction due to the selection bias resulting from using it more in migraineurs without aura than in those with aura. (2) In addition to the listed comorbidities associated with migraines affecting the risk of a stroke in this study, some factors deserve special consideration. The lifestyle and behavior patterns of migraine patients are also important factors affecting the risk of a stroke. For example, smoking and oral contraceptive pill use are known major contributors to the high risk of strokes in migraineurs [24,53]. However, we did not include these as comorbid factors that could have affected the outcome. The association of these behaviors with seeking acupuncture treatment also remains unclear. The Taiwan Biobank is a large biomedical research database that has collected rich lifestyle factors and genetic variant data from more than 130,000 participants and can be linked with the NHIRD [54,55]. Further analysis on this basis is warranted in the future. (3) In Taiwan, acupuncture treatments provided by a small number of traditional Chinese medicine doctors are self-paid and, therefore, not covered by the NHI. If patients sought these services, bias may have been introduced in our data analysis. (4) Clinical indicators are very important for evaluating the efficacy of acupuncture. Unfortunately, since the acupoints are not recorded in the database, it is impossible to analyze the correlation between the choice of acupoints in patients with migraines and the risk of stroke. Although a number of systematic reviews have reported the benefits of acupuncture for migraines, many of the studies were considered to be of low or critically low quality based on the assessment of multiple systematic review (AMSTAR) criteria [56]. We believe that rigorous large-scale prospective clinical trials should be conducted in the future. In addition, further animal experiments are needed to elucidate the mechanism through which acupuncture achieves its efficacy.

## 5. Conclusions

This 19-year-long retrospective study showed that migraineurs who received acupuncture treatment had significantly lower stroke risks, after adjustment for covariates, compared with migraineurs who did not receive acupuncture treatment. These findings support the benefit of using appropriate acupuncture treatment in patients with migraines to prevent long-term stroke risk. These results may help inform clinicians and public health policymakers.

## Figures and Tables

**Table 1:** Characteristics of new migraine patients who did or did not receive acupuncture.

	Migraine without Acupuncture		Migraine with Acupuncture		
	N = 1354		N = 1354		
Characteristic	n	%	n	%	SMD
Sex					
female	967	71.42	992	73.26	0.041
male	387	28.58	362	26.74	0.041
Age, year					
20–39	428	31.61	440	32.50	0.019
40–59	582	42.98	651	48.08	0.102
>59	344	25.41	263	19.42	0.144
mean, (SD)	48.25	15.44	46.67	14.24	0.107
Urbanization level ^‡^					
Low	774	57.16	780	57.61	0.009
Medium	464	34.27	463	34.20	0.002
High	116	8.57	111	8.20	0.013
Occupation					
Office worker	562	41.51	597	44.09	0.052
Laborer	581	42.91	564	41.65	0.025
Others ^&^	211	15.58	193	14.25	0.037
Income ^†^					
<20,000	950	70.16	925	68.32	0.04
20,000–39,999	290	21.42	322	23.78	0.057
>39,999	114	8.42	107	7.90	0.019
Comorbidities					
Diabetes mellitus	110	8.12	101	7.46	0.025
Hypertension	372	27.47	334	24.67	0.064
Hyperlipidemia	185	13.66	183	13.52	0.004
Coronary artery disease	174	12.85	155	11.45	0.043
Head injury	39	2.88	44	3.25	0.021
Parkinson’s disease	8	0.59	11	0.81	0.027
Chronic kidney disease	70	5.17	59	4.36	0.038
Mental disorders	603	44.53	592	43.72	0.016
Medication					
0–1	159	11.74	143	10.56	0.038
2–3	703	51.92	709	52.36	0.009
>3	492	36.34	502	37.08	0.015
Mean follow-up years, (SD)	9.71	7.41	13.77	6.01	0.602

Chi-square test; *t*-test. SMD: standardized mean difference. A standardized mean difference of 0.1 or less indicates a negligible difference. ^†^ New Taiwan Dollar (NTD), 1 NTD is equal to 0.03 USD. ^‡^ The urbanization level was divided by the population density of the residential area into 3 levels, where level 1 was the most urbanized and level 3 was the least urbanized. ^&^ Other occupation categories included those who were primarily retired, unemployed, and low-income populations.

**Table 2:** Hazard ratios (HRs), and 95% confidence intervals (CIs) of strokes with covariates among patients with migraines in multivariable Cox proportional hazards regression.

	Stroke						
Variables	n	PY	IR	cHR	(95% CI)	aHR ^†^	(95% CI)
Migraine without acupuncture	636	13,145	48.38	1.00	(Reference)	1.00	(Reference)
Migraine with acupuncture	387	18,644	20.76	0.47	(0.41, 0.53) ***	0.4	(0.35, 0.46) ***
Sex							
female	721	23,767	30.34	1.00	(Reference)	1.00	(Reference)
male	302	8022	37.65	1.2	(1.05, 1.37) **	1.02	(0.88, 1.17)
Age, year							
20–39	147	12,950	11.35	1.00	(Reference)	1.00	(Reference)
40–59	478	14,875	32.13	2.65	(2.2, 3.18) ***	2.55	(2.1, 3.09) ***
>59	398	3964	100.41	6.71	(5.54, 8.12) ***	4.93	(4, 6.09) ***
Urbanization level ^‡^							
Low	577	18,627	30.98	1.00	(Reference)	1.00	(Reference)
Medium	362	10,573	34.24	1.08	(0.95, 1.23)	1.03	(0.9, 1.18)
High	84	2589	32.44	1.03	(0.82, 1.3)	0.91	(0.72, 1.14)
Occupation							
Office worker	343	15,433	22.23	1.00	(Reference)	1.00	(Reference)
Laborer	511	12,076	42.32	1.77	(1.54, 2.03) ***	1.32	(1.1, 1.58) **
Others ^&^	169	4280	39.49	1.67	(1.39, 2.01) ***	1.26	(1, 1.58) *
Income ^#^							
<20,000	752	21,057	35.71	1.00	(Reference)	1.00	(Reference)
20,000–39,999	196	8037	24.39	0.71	(0.61, 0.84) ***	1	(0.82, 1.21)
>39,999	75	2694	27.84	0.8	(0.63, 1.01)	0.8	(0.6, 1.06)
Comorbidities							
Diabetes mellitus	115	1735	66.27	1.88	(1.55, 2.28) ***	1.14	(0.93, 1.41)
Hypertension	409	6008	68.07	2.48	(2.18, 2.81) ***	1.4	(1.22, 1.61) ***
Hyperlipidemia	194	3653	53.11	1.67	(1.43, 1.95) ***	1.09	(0.92, 1.3)
Coronary artery disease	199	2590	76.83	2.32	(1.98, 2.71) ***	1.32	(1.11, 1.56) **
Head injury	37	832	44.48	1.32	(0.95, 1.83)	1.66	(1.19, 2.31) **
Parkinson’s disease	15	104	144.25	3.5	(2.1, 5.83) ***	3.34	(1.98, 5.62) ***
Chronic kidney disease	66	1075	61.42	1.71	(1.33, 2.19) ***	1.06	(0.82, 1.37)
Mental disorders	525	13,124	40.00	1.43	(1.27, 1.62) ***	1.47	(1.29, 1.68) ***
Medication							
0–1	214	1524	140.45	1.00	(Reference)	1.00	(Reference)
2–3	522	16,412	31.81	0.26	(0.22, 0.3) ***	0.18	(0.15, 0.21) ***
>3	287	13,854	20.72	0.17	(0.15, 0.21) ***	0.11	(0.09, 0.13) ***

PY: person-years; IR: incidence rate per 1000 person-years; cHR: crude hazard ratio; aHR: adjusted hazard ratio; ^†^ adjusted by sex, age, urbanization level, occupation, income, comorbidities, and medication; *: *p*-value < 0.05; ** *p* < 0.01, *** *p* < 0.001 ^#^ New Taiwan Dollar (NTD), 1 NTD is equal to 0.03 USD. ^‡^ The urbanization level was divided by the population density of the residential area into 3 levels, in which level 1 was the most urbanized and level 3 was the least urbanized. & Other occupation categories included those who were primarily retired, unemployed, and low-income populations.

**Table 3:** Incidence rates, hazard ratios (HRs), and 95% confidence intervals (CIs) of strokes for migraine patients who did and did not receive acupuncture treatment in different stratifications.

	Migraine without Acupuncture			Migraine with Acupuncture								
Variables	N	PY	IR	n	PY	IR	cHR	(95% CI)	*p*-Value	aHR ^†^	(95% CI)	*p*-Value
Sex												
female	450	9789	45.97	271	13,979	19.39	0.46	(0.39, 0.53) ***	<0.001	0.37	(0.32, 0.43) ***	<0.001
male	186	3356	55.42	116	4665	24.87	0.5	(0.4, 0.63) ***	<0.001	0.32	(0.25, 0.41) ***	<0.001
Age, year												
20–39	94	5898	15.94	53	7052	7.52	0.49	(0.35, 0.69) ***	<0.001	0.32	(0.22, 0.46) ***	<0.001
40–59	295	5635	52.35	183	9240	19.81	0.41	(0.34, 0.5) ***	<0.001	0.35	(0.29, 0.42) ***	<0.001
>59	247	1612	153.26	151	2352	64.20	0.5	(0.41, 0.62) ***	<0.001	0.37	(0.3, 0.46) ***	<0.001
Urbanization level ^‡^												
Low	368	7747	47.50	209	10,880	19.21	0.44	(0.37, 0.53) ***	<0.001	0.33	(0.28, 0.39) ***	<0.001
Medium	218	4280	50.93	144	6293	22.88	0.49	(0.4, 0.61) ***	<0.001	0.38	(0.3, 0.47) ***	<0.001
High	50	1118	44.73	34	1471	23.11	0.57	(0.37, 0.88) *	0.0113	0.84	(0.52, 1.33)	0.4496
Occupation												
Office worker	214	6442	33.22	129	8991	14.35	0.46	(0.37, 0.58) ***	<0.001	0.3	(0.23, 0.38) ***	<0.001
Laborer	315	4907	64.20	196	7169	27.34	0.47	(0.4, 0.57) ***	<0.001	0.42	(0.35, 0.51) ***	<0.001
Others ^&^	107	1797	59.55	62	2483	24.97	0.47	(0.35, 0.65) ***	<0.001	0.36	(0.26, 0.51) ***	<0.001
Income ^#^												
<20,000	471	8692	54.19	281	12,365	22.73	0.47	(0.4, 0.54) ***	<0.001	0.36	(0.31, 0.43) ***	<0.001
20,000–39,999	117	3285	35.62	79	4752	16.62	0.5	(0.37, 0.66) ***	<0.001	0.34	(0.25, 0.47) ***	<0.001
>39,999	48	1168	41.10	27	1527	17.69	0.48	(0.3, 0.76) **	0.0021	0.29	(0.16, 0.52) ***	<0.001
Comorbidities												
Diabetes mellitus												
No	566	12,520	45.21	342	17,534	19.50	0.47	(0.41, 0.54) ***	<0.001	0.37	(0.32, 0.43) ***	<0.001
Yes	70	625	111.91	45	1110	40.55	0.43	(0.29, 0.62) ***	<0.001	0.26	(0.16, 0.4) ***	<0.001
Hypertension												
No	377	10,918	34.53	237	14,863	15.95	0.49	(0.42, 0.58) ***	<0.001	0.39	(0.33, 0.46) ***	<0.001
Yes	259	2227	116.28	150	3781	39.67	0.41	(0.34, 0.5) ***	<0.001	0.3	(0.24, 0.37) ***	<0.001
Hyperlipidemia												
No	523	11,739	44.55	306	16,397	18.66	0.46	(0.4, 0.53) ***	<0.001	0.34	(0.3, 0.4) ***	<0.001
Yes	113	1406	80.36	81	2247	36.05	0.51	(0.38, 0.68) ***	<0.001	0.39	(0.29, 0.54) ***	<0.001
Coronary artery disease												
No	520	12,054	43.14	304	17,145	17.73	0.45	(0.39, 0.52) ***	<0.001	0.35	(0.3, 0.41) ***	<0.001
Yes	116	1091	106.32	83	1499	55.36	0.59	(0.45, 0.79) ***	<0.001	0.39	(0.29, 0.53) ***	<0.001
Head injury												
No	611	12,875	47.46	375	18,082	20.74	0.48	(0.42, 0.54) ***	<0.001	0.37	(0.32, 0.42) ***	<0.001
Yes	25	270	92.58	12	562	21.36	0.29	(0.15, 0.58) ***	<0.001	0.12	(0.04, 0.41) ***	<0.001
Parkinson’s disease												
No	628	13,131	47.83	380	18,555	20.48	0.47	(0.41, 0.53) ***	<0.001	0.37	(0.32, 0.42) ***	<0.001
Yes	8	15	548.01	7	89	78.31	0.33	(0.12, 0.92) *	0.0336	0	(0, 0)	0.9997
Chronic kidney disease												
No	596	12,701	46.93	361	18,014	20.04	0.47	(0.41, 0.53) ***	<0.001	0.37	(0.32, 0.42) ***	<0.001
Yes	40	444	90.00	26	630	41.26	0.53	(0.32, 0.87) *	0.012	0.32	(0.17, 0.59) ***	<0.001
Mental disorders												
No	325	7615	42.68	173	11,049	15.66	0.4	(0.34, 0.49) ***	<0.001	0.3	(0.24, 0.36) ***	<0.001
Yes	311	5530	56.24	214	7594	28.18	0.55	(0.46, 0.65) ***	<0.001	0.44	(0.37, 0.52) ***	<0.001
Medication												
0–1	137	371	368.79	77	1152	66.83	0.32	(0.24, 0.43) ***	<0.001	0.35	(0.26, 0.47) ***	<0.001
2–3	344	6425	53.54	178	9986	17.82	0.37	(0.31, 0.45) ***	<0.001	0.38	(0.31, 0.45) ***	<0.001
>3	155	6348	24.42	132	7505	17.59	0.72	(0.57, 0.91) **	0.0064	0.57	(0.45, 0.73) ***	<0.001

PY: person-years; IR: incidence rate per 1000 person-years; cHR: crude hazard ratio; aHR: adjusted hazard ratio; ^†^ adjusted by sex, age, urbanization level, occupation, income, comorbidities, and medication; *: *p*-value < 0.05; ** *p* < 0.01, *** *p* < 0.001. ^#^ New Taiwan Dollar (NTD), 1 NTD is equal to 0.03 USD. ^‡^ The urbanization level was divided by the population density of the residential area into 3 levels, in which level 1 was the most urbanized and level 3 was the least urbanized. ^&^ Other occupation categories included those who were primarily retired, unemployed, and low-income populations.

**Table 4:** Incidence rates, hazard ratios, and confidence intervals of ischemic and hemorrhagic strokes in patients with migraines who did and did not receive acupuncture treatment.

	Migraine without Acupuncture			Migraine with Acupuncture								
Variables	n	PY	IR	n	PY	IR	cHR	(95% CI)	*p*-Value	aHR ^†^	(95% CI)	*p*-Value
Ischemic stroke	621	13,227	46.95	379	18,656	20.32	0.47	(0.42, 0.54) ***	<0.001	0.41	(0.36, 0.46) ***	<0.001
Hemorrhagic stroke	73	13,421	5.44	46	18,793	2.45	0.46	(0.32, 0.67) ***	<0.001	0.36	(0.25, 0.53) ***	<0.001

PY: person-years; IR: incidence rate per 1000 person-years; cHR: crude hazard ratio; aHR: adjusted hazard ratio; ^†^ djusted by sex, age, urbanization level, occupation, income, comorbidities, and medication; *** *p* < 0.001.

## Data Availability

The data in this study are available to other researchers upon request.
